# Efficient Conversion of Cane Molasses Towards High-Purity Isomaltulose and Cellular Lipid Using an Engineered *Yarrowia lipolytica* Strain in Fed-Batch Fermentation

**DOI:** 10.3390/molecules24071228

**Published:** 2019-03-28

**Authors:** Zhi-Peng Wang, Qin-Qing Wang, Song Liu, Xiao-Fang Liu, Xin-Jun Yu, Yun-Lin Jiang

**Affiliations:** 1 Key Laboratory of Sustainable Development of Polar Fishery, Ministry of Agriculture and Rural Affairs, Yellow Sea Fisheries Research Institute, Chinese Academy of Fishery Sciences, Qingdao 266071, Shandong, China; spirit87@163.com (Z.-P.W.); Liuxiaofang@ysfri.ac.cn (X.-F.L.); 2 Guangdong Provincial Key Laboratory of Marine Resources and Coastal Engineering, School of Marine Sciences, Sun Yat-Sen University, Guangzhou 510006, Guangdong, China; jiangyl7@mail2.sysu.edu.cn; 3 Southern Laboratory of Ocean Science and Engineering (Guangdong, Zhuhai), Zhuhai 519000, China; 4 Development & Reform Bureau, West Coast New Area, Qingdao 266000, Shandong, China; shoushoudecuzi@163.com; 5 Key Laboratory of Bioorganic Synthesis of Zhejiang Province, College of Biotechnology and Bioengineering, Zhejiang University of Technology, Hangzhou 310014, Zhejiang, China; xjyu@zjut.edu.cn

**Keywords:** cane molasses, isomaltulose, *Yarrowia lipolytica*, lipid

## Abstract

Cane molasses is one of the main by-products of sugar refineries, which is rich in sucrose. In this work, low-cost cane molasses was introduced as an alternative substrate for isomaltulose production. Using the engineered *Yarrowia lipolytica*, the isomaltulose production reached the highest (102.6 g L^−1^) at flask level with pretreated cane molasses of 350 g L^−1^ and corn steep liquor of 1.0 g L^−1^. During fed-batch fermentation, the maximal isomaltulose concentration (161.2 g L^−1^) was achieved with 0.96 g g^−1^ yield within 80 h. Simultaneously, monosaccharides were completely depleted, harvesting the high isomaltulose purity (97.4%) and high lipid level (12.2 g L^−1^). Additionally, the lipids comprised of 94.29% C_16_ and C_18_ fatty acids, were proved suitable for biodiesel production. Therefore, the bioprocess employed using cane molasses in this study was low-cost and eco-friendly for high-purity isomaltulose production, coupling with valuable lipids.

## 1. Introduction

Cane molasses is one of the main by-products of sugar refineries, which contains saccharides (primarily sucrose, glucose and fructose) and a small amount of nitrogenous compounds, vitamins, and trace metal elements as well as colloids [1,2]. Sugarcane grows worldwide and mainly distributes in Brazil, India, and China. The molasses production in China is around 400 million tons per year, yet, large volumes of waste molasses are simply discharged, contributing to severe environmental pollution [3]. Cane molasses can be used as an available source of quick energy for animal feed or feed supplement [4]. However, livestock frequently suffers from some nervous symptoms and blindness caused by molasses toxicity [5]. Given that available ingredients enrich in cane molasses, nowadays, it is increasingly utilized as an alternative feedstock for microbial fermentation after a pretreatment or as the raw material. Different high value-added metabolites have been harvested via microbial fermentation from molasses, such as astaxanthin [6], 5-hydroxymethylfurfural [7], ethanol [8,9], organic acids [3,10,11,12], and enzymes [13].

As a functional sweetener from microbial fermentation, isomaltulose has attracted extensive attention. Isomaltulose (or palatinose) is a structural isomer of sucrose, sharing similar sweet sense and fine taste with sucrose. It has been approved that a safer sucrose substitute with some advantages, including higher stability, lower digestibility, lower glycemic index and more tooth-friendly [14]. Isomaltulose can be converted from sucrose by sucrose isomerase (SIase) without any cofactors due to the less free-energy of the process [15]. Several SIase-producing microbes were adopted for isomaltulose production, such as *Pantoea dispersa* [16], *Erwinia* spp. [17], *Enterobacter* spp. [18], and *Serratia plymuthica* [19]. However, they have not been considered to synthesize isomaltulose commercially for lack of genetic background in food-grade level [2,20].

Recently, heterologous expression of SIases in non-pathogenic or food-grade strains, such as *Saccharomyces cerevisiae* (*S. cerevisiae*), *Yarrowia lipolytica* (*Y. lipolytica*), *Bacillus subtili* (*B. subtili*), and *Lactococcus lactis* (*L. lactis*), has been proved effective for isomaltulose production [2,18,21,22]. Noteworthily, the recombinant *Y. lipolytica* expressing SIase gene has exhibited a prominent isomaltulose yield advantage over other hosts, which meets the requirement of isomaltulose commercialization [22,23]. Since sucrose substrate takes a substantial part of the total production cost, reusing low-cost materials rich in sucrose can be a feasible way to reduce the cost.

In traditional cane molasses fermentations using sucrose as the carbon source, sucrose was hydrolyzed into monosaccharides and utilized by microorganisms. However, the strain used in this study can just utilize monosaccharides, leaving sucrose not hydrolyzed for no sucrase generating. The high sucrose content in cane molasses makes it a promising substrate for isomaltulose production. On the other hand, the synthesis of isomaltulose from cane molasses can greatly improve the value of cane molasses. In this study, pretreated cane molasses (PCM) was used as the only carbon source, and corn steep liquor (CSL) was selected as substrate to replace yeast extract for the recombinant *Y. lipolytica* S47, which is capable of expressing SIase and transforming monosaccharides to lipids as shown. To further develop an economical fermentation and recycling efficiently by-products using strain S47, fed-batch fermentation was also conducted.

## 2. Results and Discussion

### 2.1. Isomaltulose Production Using PCM as Sole Carbon Source

Sucrose in cane molasses can be hydrolyzed into monosaccharides by microbial enzymes, which is believed to be suitable for microbial growth and metabolite production [8,13]. In this study, to develop a cost-effective fermentation, PCM was used for cells growth and isomaltose synthesis. As shown in Table 1, isomaltulose production increased as the activity of SIase secreted from strain S47 improved, based on the increase of PCM concentration. A maximal isomaltulose concentration (96.7 g L^−1^) was obtained with the SIase activity of 3.6 U mL^−1^ at 350 g L^−1^ PCM, and the yield achieved 0.96 (g g^−1^). Although the SIase activity (3.6 U mL^−1^) was limited, which is lower than 5.2, 7.4 and 49.3 U mL^−1^ [2,23,24], this yield is identical to the same strain using sucrose as a substrate [23]. The results proved cane molasses as a suitable substrate for isomaltulose synthesis.

**Figure 1:**
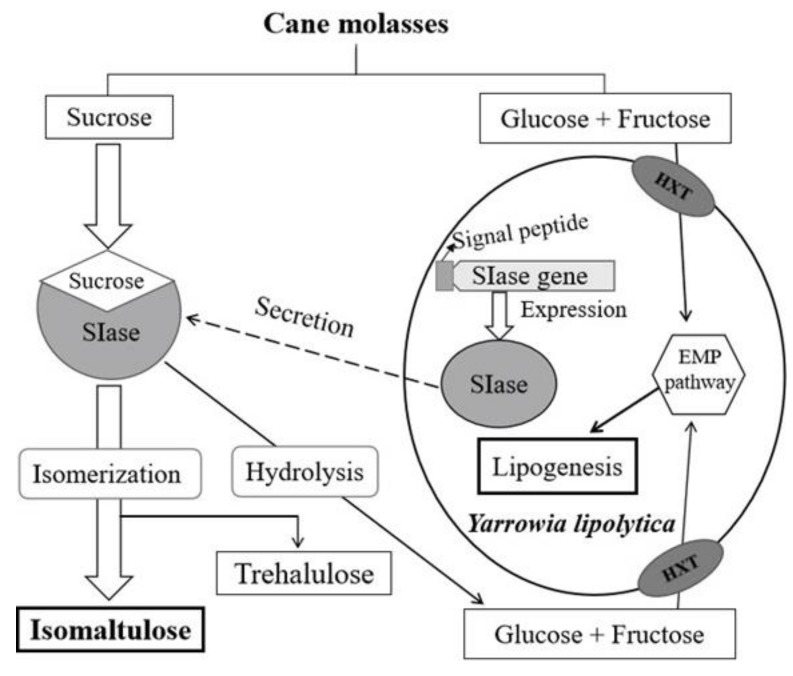
The flow of different components in cane molasses during the fermentation process by the engineered strain *Y. lipolytica* S47 (HXT-hexose transporter).

In addition, as strain S47 cells grew to the peak, intracellular lipids continuously accumulated and reached 5.3 g L^−1^, which accounted for 43.1% (*w/w*) of cell dry weight, respectively (Table 1). When PCM concentration exceeded 350 g L^−1^, isomaltulose and lipid productions both declined significantly, so did isomaltulose yield and others, except for residual sugar content (Table 1). This might be the result of cell dehydration and growth inhibition triggered by high PCM concentration, leading to suppressed expression of SIase and several crucial lipid-synthesizing enzymes, such as delta-9 stearoyl-CoA desaturase and acetyl-CoA carboxylase [2,25]. Generally, all the components in cane molasses can be consumed by the yeast. The flow of sucrose was directed into SIase-catalyzing isomaltulose synthesis, the byproducts of the SIase-catalyzing reaction were utilized by the yeast; monosaccharides in cane molasses were consumed for cell growth and lipid production (Figure 1). However, the residual sugar concentration (8.6 g L^−1^) presented might infer that yeast extract is not desirable nitrogen source in the medium. Therefore, an alternative nitrogen source will be needed to solve the residual sugar.

### 2.2. Effect of CSL on Enhancement of Isomaltulose and Lipid Production

CSL generally contains abundant nutritional ingredients. It has been evaluated to be favorable for microbial growth and synthesis of natural products [26,27]. To further facilitate the conversion of PCM effectively to isomaltulose and lipid production, CSL was utilized as organic nitrogen to avoid the expensive yeast extract. Cell growth and fermentation at different concentrations of CSL media are shown in Table 2. It showed that most sucrose in PCM was converted to isomaltulose (102.6 g L^−1^) with 0.96 g g^−1^ yield, followed with enhanced SIase activity by 20.5% and biomass by 11.8% at the optimal CSL concentration (1.0 g L^−1^) compared with those in control (Table 2). Meanwhile, a distinct consumption of residual sugar was reduced by 73.3%. As a result, sugar reduction contributed to improving isomaltulose production by 5.5%, and lipids (7.2 g L^−1^) by 35.8% with 44.7% (*w/w*) content (Table 2). Enhancements of CSL for isomaltulose and lipid co-production indicated that CSL is more suitable for strain S47 growth to produce target products than yeast extract, and it could act as an economic alternative for yeast extract.

To date, CSL has been also employed to produce diverse metabolites [26,28,29]. The result in table 2 showed that CSL played important roles in enhancing biomass generation and isomaltulose production due to the high secreted SIase activity, which is consistent with their positive changes in CSL-added media [2,27]. Nitrogen sources affect cell growth and metabolites formation during microbial fermentation. The components of amino acids, vitamins and trace elements in CSL may help boost initial biomass formation, while amino acids and/or biotin could also start releases of related enzymes for synthesizing metabolites [29]. The reports would probably account for improvements of biomass, SIase activity and isomaltulose production in this study. Besides, during CSL fermentation, intermediates (e.g., citric acid and poly malic acid) required for lipid accumulation have been enhanced in previous studies resulting from overexpression of crucial enzymes, i.e., citric acid synthetase (CS), malic enzymes (ME) and pyruvate carboxylase (PYC) [26]. We deduce that these enzymes (CS, ME and PYC) would perform at higher levels and synthesize more citric acid and poly malic acid in this study, further facilitating intracellular lipid accumulation. Moreover, significant sugar consumption and transformation during CSL fermentation suggest monosaccharides might flow mainly to lipid accumulation and cell growth, and a small share for isomaltulose production.

### 2.3. Fed-Batch Fermentation for Isomaltulose and Lipid

**Figure 2:**
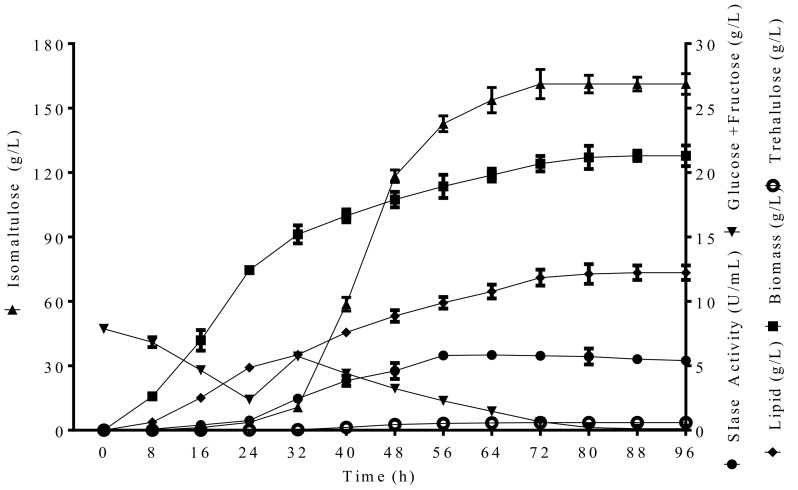
Time course of PCM biotransformation towards isomaltulose and lipid using CSL substrate by *Y. lipolytica* S47 in a 10-L bioreactor. Data are given as means ± standard deviation, n = 3.

High osmotic pressure strongly affects product biosynthesis of *Y. lipolytica* cells [30]. To reduce high osmotic pressure in the optimized medium and to improve isomaltulose production, fed-batch fermentation was conducted using a two-stage bioprocess in a 10-L bioreactor. Figure 2 shows that cell growth, SIase activity and isomaltulose production were all detected to increase slowly when the process was close to 32 h in the first stage. Remarkably, during the second stage after 200 g L^−1^ PCM supplemented, cell growth and isomaltulose concentration were both switched to increase and achieved the maximal values of 21.3 g L^−1^ and 161.2 g L^−1^ at 80 h accompanied by higher SIase activity (5.7 U mL^−1^), respectively (Figure 2). Generally, the higher yield and higher production are the key factors of saving cost. The yield derived from PCM and CSL in the bioreactor maintained 0.96 g g^−1^, which reaches the same level from engineered *Y. lipolytica* strains both expressing SIase using sucrose as the substrate [23,24]. The isomaltulose concentration (161.2 g L^−1^) was also evidently higher compared with those (36, <4, <33.5 g L^−1^) from strains *L. lactis*, *S. cerevisiae* and *S.* *plymuthica* using sucrose or molasses as the substrate, respectively (Table 3). It indicates that sucrose in PCM was almost converted completely at the end of the fermentation. In fact, Li et al. (2013) revealed that high SIase activity (48 U mL^−1^) was achieved by a recombinant *Escherichia coli* expressing SIase using cane molasses and CSL substrates, while it lacks isomaltulose concentration and yield [31].

In addition, lipids accumulated to 12.2 g L^−1^ and the content reached 57.3% (*w*/*w*) during the two–stage fermentation (Figure 2), which is enhanced markedly than 7.2 g L^−1^ production at flask level above and that (8.1 g L^−1^) produced by the same strain from sucrose [23]. The lipid content (57.3%) obtained is much higher than those from *Rhodotorula kratochvilovae* (25%, *w/w*) and engineered *Ashbya gossypii* (38.25%, *w/w*) using cane molasses, respectively [32,33]. Strain S47 can be regarded as an oleaginous yeast due to the lipid content over 20% [26]. It also exhibits a significant advantage over liquid contents (23.4–49.6%) of other specially engineered *Y. lipolytica* strains and *Aureobasidium melanogenum*, and even co-cultured strains using other substrates [25,26,34,35]. Consequently, lipid accumulation coupled with isomaltulose production by strain S47 was demonstrated to be economical and valuable.

It is noteworthy that original monosaccharides and bits of them produced by SIase catalysis from strain S47 were depleted and only small amounts of trehalulose were detected, leading to produce a high isomaltulose purity of 97.4%. This purity is comparable with that (97.8%) by strain S47 using sucrose [23]. In fact, isomaltulose is difficult to separate completely from a fermented mixture including trehalulose, glucose, fructose and residual sucrose [22,27]. However, high-value lipid accumulation in this work was testified to benefit isomaltulose purity due to the consumption of undesirable by-products. The results suggest that high-purity isomaltulose can be achieved successfully via coupling with considerable lipid accumulation by the food-grade strain *Y. lipolytica* S47 using PCM and CSL substrates in fed-batch fermentation, thus, avoiding the effect of residual sugar on isomaltulose crystal.

### 2.4. Fatty Acids Composition of Intracellular Lipids and Biodiesel Production

After transmethylated fatty acids were detected, the main fatty acids of intracellular lipids were C_14:0_, C_16:0_, C_16:1_, C_18:0_, C_18:1_ and C_18:2_, especially C_18:1_ (45.14%) (Table 4). This is similar to those produced by *Rhodotorula glutinis* TR29 cultivated in molasses medium, with producing 63.5% C_18:1_ fatty acid [36]. Moreover, many microbes such as *A. melanogenum*, *Rhodosporidium toruloides*, and other engineered *Y. lipolytica* strains are capable of generating fatty acids of intracellular lipids that primarily contain C_16_–C_18_ and the largest share of C_18:1_ using other substances [25,26]. Indeed, 94.29% of fatty acids of lipids from strain S47 cells in this study were C_16_ and C_18_. It reveals that lipids accumulated in *Y. lipolytica* S47 cells from cane molasses are suitable for biodiesel production that requires C_16_ and C_18_ fatty acids [36,37].

Biodiesel was prepared from the transesterification of obtained intracellular lipids. In this work, the conversion rate of lipids into biodiesel reached near 83.2% (data not shown), which is comparable with others (85–86.7%) [25,38], indicating that intracellular lipids produced from *Y. lipolytica* S47 cells is a promising feedstock for biodiesel production.

## 3. Materials and Methods

### 3.1. Strain and Cane Molasses Fermentation

The recombinant *Y. lipolytica* S47 was previously constructed and cultivated as seed culture in YPD medium (yeast extract 1%, peptone 2%, and dextrose 2%) [23]. The promoter for expression of SIase gene was constitutive, thus, recombinant SIase can be secreted extracellularly without any induction [23]. Furthermore, strain S47 was derived from the typical oleaginous yeast *Y. lipolytica* ACA-DC 50109, which can transform extracellular sugars into cellular lipid [23]. Cane molasses was obtained from a sugar refinery in Guangxi and it was pretreated as described in the methods [24]. The composition of pretreated cane molasses (PCM) consisted of sucrose (30.55%, *w/v*), glucose (5.71%) and fructose (7.78%). Glucose potassium phosphate buffer (GPPB) medium with added different concentrations of PCM (200, 250, 300, 350, 400 g L^−1^) instead of glucose was optimized to improve isomaltulose production by strain S47 at 30 °C for 96 h, pH was adjusted to 6.0 [23]. By the action of phosphate buffer in the medium, pH can be stable.

### 3.2. CSL Optimization for Enhancement of Isomaltulose and Lipid Co-Production

Corn steep liquor (CSL) was derived from a local corn processing facility, which was used as a substitute for yeast extract in the fermentation medium. The fermentation was performed at different concentrations of CSL (0.5, 1.0, 1.5 g L^−1^) to enhance isomaltulose production and purity, together with lipid synthesis compared to those from strain S47 fermentation using 0.5 g L^−1^ yeast extract. pH was adjusted to 6.0.

### 3.3. Fed-Batch Fermentation in a 10-L Fermentor

Fermentation was scaled up in a 10-L Biostat fermentor (B. Braun Biotech International, Melsungen, Germany) with the obtained medium (6.0 L), which contained optimized PCM (350 g L^−1^) and CSL (1.0 g L^−1^). After inoculation (5.0%, *v/v*), the strain fermented to secrete SIase for isomaltulose synthesis and express some enzymes like ATP citrate lyase to accumulate intracellular lipids under the conditions of aeration rate (50 L min^−1^), rotate speed (300 rpm), and temperature (30 °C) [23]. Fed-batch fermentation was carried out by adding 200 g L^−1^ PCM at the initial 32 h of fermentation. During bioprocess, samples (50 mL) were taken at intervals of 7 h to detect isomaltulose and lipid contents, SIase activity and monosaccharides as well as biomass. pH was controlled at 6.0.

### 3.4. Enzyme Assay and Determination for Isomaltulose and Lipid Contents, Residual Sugar

The fermented broth from strain S47 was centrifuged at 5000× *g* to obtain the supernatant, and SIase activity was measured according to previous methods [16,22]. Briefly, the supernatant was added to a sucrose solution (100 g L^−1^) that dissolved in phosphate buffer (50 mM and pH 6.0) in a ratio of 1:1; the mixture was then incubated at 30 °C for 10 min. The process was terminated through boiling for 10 min and centrifuged (10,000× *g*, 20 min) to eliminate denatured proteins. Finally, the new supernatant was filtered through 0.22 μm membrane and properly diluted for high-performance liquid chromatography (HPLC) analysis using Agilent 1200 system (Agilent Technologies, Palo Alto, CA, USA). One unit (U) of enzyme activity was calculated as the SIase amount responsible for the release of 1.0 μmol isomaltulose per min at 30 °C and pH 6.0 [23]. The fermented supernatant was boiled before mixing as the control.

The contents of isomaltulose, trehalulose, residual glucose and fructose in fermentation supernatant were also determined using HPLC after membrane (0.22 μm) filtration. Biomass and quantification of intracellular lipids were gravimetrically detected [25]. All tests above were performed in triplicate.

### 3.5. Determination of Lipid Composition and Preparation for Biodiesel

Lipid was extracted and fatty acid esters were determined using gas chromatography (GC) [26]. 1 μL of sample was injected in a 10:1 split mode at 275 °C, using helium as the carrier gas at a flow rate of 1 mL/min. The GC oven temperature was held at 150 1C for 1 min and ramped to 230 °C (rate: 15 °C /min, hold: 2 min) for a total run time of approximate 13.5 min. Different fatty acid methyl esters were identified and characterized using the authentic fatty acid methyl ester (FAME) standards. Biodiesel was prepared and detected using H_2_SO_4_ solution (in methanol) through thermocatalytic treatment as described in previous research [25].

### 3.6. Statistical Analysis

The results obtained above were subjected to a one-way analysis of variance (ANOVA) using SPSS 22.0 software (SPSS Inc., Chicago, MI, USA), and presented as the mean ± standard deviation. Statistically significant differences between groups were showed as *p* < 0.05 (*) and *p* < 0.01 (**).

## 4. Conclusions

An efficient, economical strategy to produce isomaltulose from cane molasses was established. During the fed-batch fermentation, the maximal isomaltulose concentration achieved 161.2 g L^−1^ and yielded 0.96 g g^−1^. Monosaccharides were completely consumed and transformed into intracellular lipids and biomass, which resulted in a high isomaltulose purity of 97.4% and 12.2 g L^−1^ lipids. The lipids produced from *Y. lipolytica* S47 cells have potential as a candidate for biodiesel production. 

## Figures and Tables

**Table 1:** Isomaltulose and lipid production from PCM of different concentrations.

PCM (g L^–1^)	Isomaltulose (g L^−1^)	SIase (U mL^−1^)	Lipid Content (g L^−1^)	Biomass (g L^−1^)	Residual Sugar (g L^–1^)
200	58.6 ± 2.3	3.3 ± 0.2	3.8 ± 0.2	9.9 ± 0.7	1.3 ± 0.1
250	73.3 ± 7.2	3.3 ± 0.1	4.6 ± 0.3	11.8 ± 0.6	1.6 ± 0.1
300	88.1 ± 6.6	3.6 ± 0.1	5.2 ± 0.2	12.6 ± 1.2	1.9 ± 0.2
350	96.7 ± 4.9	3.6 ± 0.2	5.3 ± 0.3	12.3 ± 0.5	8.6 ± 0.4
400	71.9 ± 6.3	3.5 ± 0.2	4.8 ± 0.2	11.8 ± 0.9	42.8 ± 2.7

**Table 2:** Isomaltulose and lipid production with different CSL concentrations.

CSL (g L^−1^)	Isomaltulose (g L^−1^)	SIase (U mL^−1^)	Lipid Content (g L^−1^)	Biomass (g L^−1^)	Residual Sugar (g L^−1^)
Control	96.7 ± 7.2	3.6 ± 0.2	5.3 ± 0.3	12.3 ± 0.4	8.6 ± 0.5
0.5	102.6 ± 5.4	3.9 ± 0.1	6.7 ± 0.4	14.4 ± 0.8	2.3 ± 0.1
1.0	102.6 ± 4.9	4.7 ± 0.1	7.2 ± 0.3	16.1 ± 0.6	2.3 ± 0.1
1.5	102.6 ± 6.7	4.9 ± 0.2	6.2 ± 0.4	19.7 ± 0.7	2.3 ± 0.1

**Table 3:** Isomaltulose production obtained from different substrates by diverse engineered food-grade strains.

Strains	Substrate	Isomaltulose Production (g L^−1^)	Yield (g g^−1^)	Isomaltulose Proportion (%)	Other Products (g L^−1^)	References
*L. lactis*	Sucrose	36	0.72	<90	-	[18]
*S. cerevisiae*	Sucrose	<4	0.074	<10	-	[21]
*Y. lipolytica*	Sucrose	572.1	0.96	97.8	Lipid, 8.1	[23]
*Y. lipolytica*	Sucrose	620.7	0.96	-	-	[24]
*S. plymuthica*	Molasses	<33.5	0.84	80.4	-	[20]
*B. subtilis*	Molasses	212.6	0.92	<92.4	-	[2]
*Y. lipolytica*	Molasses	161.2	0.96	97.4	Lipid, 12.2	This study

”-” represented that no other products produced or the products did not get detected.

**Table 4:** The fatty acid composition of intracellular lipids from *Y. lipolytica* S47 cells.

Fatty Acids	C_14:0_	C_16:0_	C_16:1_	C_18:0_	C_18:1_	C_18:2_
Percentage (%)	5.71	16.55	12.67	6.82	45.14	13.11
